# Sex-different abnormalities in the right second to fourth digit ratio in Japanese individuals with autism spectrum disorders

**DOI:** 10.1186/s13229-015-0028-x

**Published:** 2015-06-09

**Authors:** Yasuhiro Masuya, Yuko Okamoto, Keisuke Inohara, Yukiko Matsumura, Toru Fujioka, Yuji Wada, Hirotaka Kosaka

**Affiliations:** Department of Neuropsychiatry, Faculty of Medical Sciences, University of Fukui, Fukui, 910-1193 Japan; Research Center for Child Mental Development, University of Fukui, Fukui, 910-1193 Japan; Division of Developmental Higher Brain Functions, Department of Child Development, United Graduate School of Child Development, Osaka University, Kanazawa University, Hamamatsu University School of Medicine, Chiba University and University of Fukui, Fukui, 910-1193 Japan; Department of Informatics, Graduate School of Informatics and Engineering, The University of Electro-Communications, Chofu, 182-8585 Japan

**Keywords:** Sex difference, Autism spectrum disorders, Digit ratio, Prenatal testosterone, Prenatal estrogen, Etiology

## Abstract

**Background:**

The prevalence of autism spectrum disorders (ASDs) is higher in men than in women. The extreme male brain theory proposes that excessive prenatal testosterone activity could be a risk factor for ASDs. However, it is unclear whether prenatal sex hormone activity is a risk factor for women. The ratio of the length of the second to fourth digits (2D:4D) is considered to be a biomarker of the prenatal ratio of testosterone to estrogen. Therefore, this study compared the 2D:4D ratios of women with and without ASDs to determine if prenatal sex hormone activity could be a risk factor for ASDs in women.

**Methods:**

The study included 35 Japanese men with ASDs, 17 Japanese women with ASDs, 59 typically developed (TD) Japanese men, and 57 TD Japanese women. We measured digit lengths and compared the 2D:4D ratios among the four groups. We also examined the relationship between the 2D:4D ratio and the autism-spectrum quotient score of each group.

**Results:**

In our cohort, men with ASDs tended to have lower right-hand 2D:4D ratios relative to TD men. In contrast, the right 2D:4D ratios in women with ASDs were higher compared to those of TD women. No significant correlations were found between the 2D:4D ratios and the autism-spectrum quotient scores in any group. The higher right 2D:4D ratios in women could not be explained by age or full-scale intelligent quotients. This group difference was not found for the left 2D:4D or right–left 2D:4D ratios.

**Conclusions:**

We found a reverse direction of abnormality in the right 2D:4D ratio for men and women with ASDs. It has been posited that high prenatal testosterone levels lead to a lower 2D:4D ratio. However, a recent animal study showed that testosterone injection to dam leads to a higher right 2D:4D ratio especially for female offspring, which might be mediated by abnormal adipose accumulation in the fingertip. Therefore, the present findings suggest that high prenatal testosterone could be a risk factor both for Japanese men and women with ASDs, elucidating one potential etiology of ASDs in women.

## Background

Autism spectrum disorders (ASDs) are a group of neurodevelopmental disorders characterized by difficulties in social communication and interaction and restricted, repetitive patterns of behavior, interests, or activities [1]. Previous studies have shown that the prevalence ratio for ASDs is four times greater in men than in women [2, 3]. Based on such sex-biased prevalence ratios, a number of studies have investigated men-specific hormonal or genetic candidates for ASD risk factors [3–5]. However, the etiology of ASDs in women is largely unknown.

Baron-Cohen and colleagues proposed the extreme male brain (EMB) theory, in which the activities of sex hormones such as testosterone and estrogen during the prenatal period is one of the risk factors for ASDs [6–9]. In typically developing (TD) individuals, male fetuses are exposed to at least 2.5-fold higher levels of testosterone than female fetuses between weeks 8 and 24 of gestation [10]. This androgen exposure is thought to masculinize cognition, resulting in higher systemizing ability (i.e., understanding things as systems in terms of rules) and lower empathizing ability (i.e., understanding and responding to the mental states of others) [6–9]. Based on this hypothesis, excessive fetal testosterone exposure in men with ASDs causes “extreme male” cognition (superior systemizing and poorer empathizing) relative to TD men [6–9]. Indeed, a recent study demonstrated that testosterone levels in the amniotic fluid samples of mothers of male babies later diagnosed with ASDs were elevated relative to those of TD male babies [11]. However, it is unknown whether women with ASDs were exposed to high testosterone levels in utero.

Although measurement of amniotic fluid can be used to directly assess prenatal sex hormone exposure, the low prevalence of ASDs in women would require a large cohort study. As an alternative indirect measure, the ratio of the second to fourth digit length (2D:4D) is frequently used to estimate prenatal sex hormone activity. The ratio is affected by both the amount of sex hormone exposure and sex hormone sensitivity [12–15]. Lutchmaya et al. showed that the 2D:4D ratio of the right hand was negatively correlated with the ratio of testosterone to estrogen in amniotic fluid, indicating that individuals exposed to a higher testosterone:estrogen ratio tend to have lower 2D:4D ratios [12]. A mouse study also demonstrated that inactivation of androgen or estrogen receptors leads to higher or lower 2D:4D ratios, respectively [15]. If high testosterone activity (especially relative to estrogen) is a risk factor for both sexes, both men and women with ASDs should have lower 2D:4D ratios.

In support of the EMB theory, recent meta-analyses of 2D:4D ratios in subjects with ASDs have suggested that ratios are lower in individuals with ASDs compared to TD individuals [16, 17]. However, there were few women with ASDs, if any, in most of the investigations [18–27]. Thus, the 2D:4D ratios in women with ASDs were not sufficiently examined in previous studies. Only two studies examining 2D:4D ratios have involved large numbers of women with ASDs (>10) [28, 29], and neither reported a significant difference in the 2D:4D ratios between women with ASDs and TD women [28, 29], suggesting that prenatal sex hormone activity is not a risk factor for women with ASDs.

However, previous studies have shown that the prevalence ratio for ASDs differs among different races/ethnicities (e.g., the increased risk for ethnic blacks [30, 31]). One possible cause for variable prevalence ratios is different levels of prenatal sex hormone activity among races. For instance, an American study of TD individuals reported higher testosterone levels in the amniotic fluid of black women relative to white women [32]. Another group measured higher testosterone levels in the umbilical cord blood of Asian babies in China compared to Caucasian babies in the USA [33]. Furthermore, race differences have also been observed in 2D:4D ratio values [34–36] and its relationships with other indices such as sexual orientation [37] or number of children [34]. These findings indicate that prenatal sex hormone activity could be a risk factor for ASDs in women of races exposed to higher testosterone in utero, such as Asian or African women. Notably, the two previous studies examining 2D:4D ratios in women with ASDs only assessed Caucasian women (Swedish or Dutch) [28, 29].

Here, we examined the 2D:4D ratios of women and men with ASDs and TD. We hypothesized that if a higher level of prenatal testosterone activity were a risk factor for ASDs in both sexes, both women and men with ASDs should have lower 2D:4D ratios relative to their TD counterparts. We also examined the relationships between the 2D:4D ratio and individual autistic traits as measured using the autism-spectrum quotient (AQ) [38] for each group.

## Methods

### Subjects

Fifty-two Japanese individuals with ASDs (35 men and 17 women) and 139 Japanese TD individuals (71 men and 68 women) participated in the present study (Table 1). The protocol was approved by the ethics committee of the University of Fukui (Japan), and the study was conducted in accordance with the Declaration of Helsinki. Participants were excluded if they had a history of major medical or neurological illness including epilepsy or significant head trauma or a lifetime history of alcohol or drug dependence. Written informed consent was obtained from each participant following a complete explanation of the study. ASD participants’ intelligence quotient (IQ) scores were obtained using the Wechsler Adult Intelligence Scale-III (WAIS-III) [39]. We also measured AQ scores, which have been validated in a clinical sample [40], for all participants.

**Table 1:** Demographic data

	ASDs		TD	
	Men	Women	Men	Women
Number	35	17	59	57
Age	29.7 ± 7.1	25.9 ± 6.6	27.0 ± 7.9	28.5 ± 11.3
WAIS				
FSIQ	102.3 ± 15.0	98.6 ± 10.9		
vIQ	105.6 ± 17.6	99.3 ± 11.6		
pIQ	97.4 ± 14.3	98.1 ± 10.9		
AQ				
Total	33.8 ± 5.2	33.2 ± 3.9	14.1 ± 3.6	11.2 ± 4.1
Social	8.2 ± 1.9	8.3 ± 1.8	2.5 ± 1.8	1.6 ± 1.7
Attention switching	7.4 ± 1.9	7.8 ± 1.4	3.7 ± 1.7	2.9 ± 1.3
Attention to detail	5.2 ± 2.6	4.2 ± 2.0	3.5 ± 2.3	3.6 ± 2.0
Communication	7.1 ± 2.1	7.2 ± 1.7	1.6 ± 1.4	1.3 ± 1.4
Imagination	5.7 ± 2.4	5.7 ± 1.2	2.9 ± 1.7	1.8 ± 1.4

Age, IQ scores, and AQ scores are shown as mean ± SD

*ASD* autism spectrum disorder, *TD* typically developed, *WAIS* Wechsler Adult Intelligence Scale, third edition (Wechsler, 1997), *AQ* Autism-spectrum quotient

#### Individuals with ASDs

Thirty-five men with ASDs (mean age ± standard deviation [SD] = 29.7 ± 7.1 years) and 17 women with ASDs (25.9 ± 6.6 years) were recruited from the Department of Neuropsychiatry at the University of Fukui Hospital (Table 1). A psychiatrist (H.K.) diagnosed participants according to the Diagnostic and Statistical Manual of Mental Disorders [1] and the standardized criteria of the Diagnostic Interview for Social and Communication Disorders [41], which reportedly possesses good psychometric properties [42]. This instrument also contains items concerning early development and a section on activities of daily living, which provide data regarding functioning in areas other than social- and communication-related domains [41]. Full-scale IQ (FSIQ) scores were greater than 70 for all ASD participants. An independent sample *t* test revealed that there was no significant difference in FSIQ scores between men and women with ASDs (*t*(50) = 0.90, *p* = 0.374).

#### TD individuals

Seventy-one TD men and 68 TD women were recruited from the local community, including staff, students, clerical officers, and local sports squads at the University of Fukui. They were screened to exclude individuals who had a first-degree relative with an axis I disorder based on DSM-IV-TR criteria [43]. Autistic traits are considered on the spectrum regardless of diagnosis, so TD participants were limited by total AQ score (<20) to exclude TD individuals with more prominent traits. Based on the criteria, we excluded 12 TD men and 11 TD women from the data analysis. Thus, data from 59 TD men (27.0 ± 7.9 years) and 57 TD women (28.5 ± 11.3 years) were used for subsequent data analysis (Table 1).

A two-way analysis of variance (ANOVA) testing age with two levels for groups (ASDs/TD) and two levels for sexes (men/women) revealed no significant interaction between group and sex (*F*(1,164) = 2.88, *p* = 0.091), main effect of group (*F*(1,164) = 0.03, *p* = 0.960), or main effect of sex (*F*(1,164) = 0.55, *p* = 0.460). A two-way ANOVA examining total AQ scores revealed a significant main effect of group (*F*(1,164) = 826.35, *p* < 0.001) and main effect of sex (*F*(1,164) = 5.83, *p* = 0.017). However, there was no significant interaction between sex and group (*F*(1,164) = 2.68, *p* = 0.104).

### Measurement of 2D:4D and statistical analysis

The ventral surfaces of each participant’s hands were photocopied. The length of the index and annular fingers from the base to the tip were then measured in the photocopies by two independent measurers using vernier calipers with sensitivity set to 0.01 mm. In order to test the intra-observer repeatability of digit length and 2D:4D ratios, we utilized the single-score interclass correlation coefficient (ICC) and, employed a two-way mixed-effects model with an absolute-agreement definition for digit length and 2D:4D ratios, respectively. The ICCs of digit length and 2D:4D ratios of the two measurers were 0.97 and 0.74, very similar to the 0.95 and 0.75 reported in a previous study [44]. We then calculated the mean digit lengths of 2D and 4D, and the mean 2D:4D ratios between two examiners in the right and left hands of each participant. Recent studies have proposed that the difference in the 2D:4D ratios between the right and left hands (right–left 2D:4D ratio) could be a biomarker of prenatal testosterone and estrogen activity [45]. Therefore, we calculated both right–left 2D:4D ratios and separate right and left 2D:4D ratios.

All statistical analyses were performed using IBM SPSS statistics software, version 20 (IBM Corporation). We initially conducted a two-way ANOVA with two levels for group (ASDs and TD) and two levels for sex (women and men) to examine the digit length of 2D and 4D for both hands, and the right, left, and right–left 2D:4D ratios. We then performed correlation analyses to examine associations between AQ scores and 2D:4D ratios in each group.

## Results

### Sex and group differences in digit length and 2D:4D ratios

**Fig. 1:**
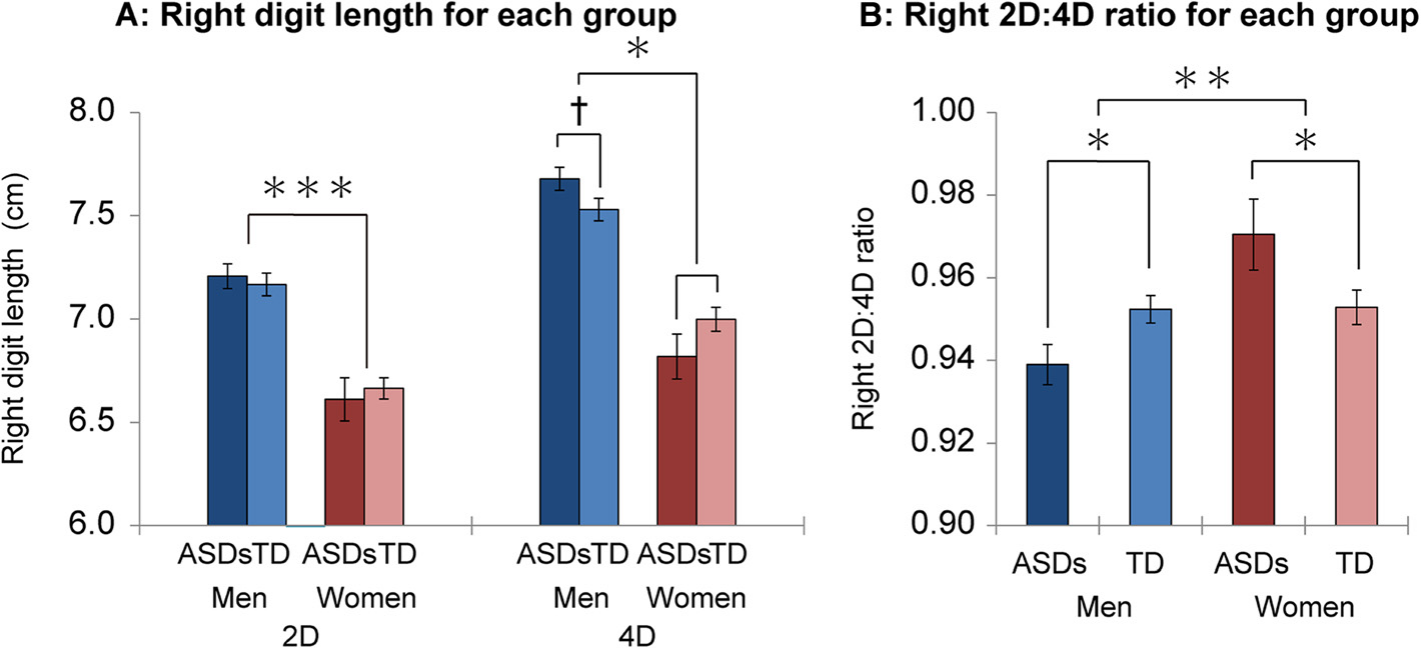
Group differences in right digit length and right 2D:4D ratio. Panels **a** and **b** show group differences in right digit length and right 2D:4D ratios, respectively. *Error bars* indicate standard errors of the means. †*p* < 0.10, *p < 0.05, **p < 0.01, ***p < 0.001

Table 2 shows the means and SDs for digit length and 2D:4D ratios for each group. For individual digit length, two-way ANOVAs examining the right 4D according to group and sex revealed a significant interaction between sex and group (*F*(1,146) = 5.27, *p* = 0.023) and main effect of sex (*F*(1,146) = 93.06, *p* < 0.001), while there was no significant main effect of group (*F*(1,146) = 0.05, *p* = 0.825). Post hoc pair-wise comparisons with the Bonferroni correction revealed that men with ASDs tended to have marginally significant longer right 4D relative to TD men (*p* < 0.10, Cohen’s *d* = 0.37 [46]), while no significant difference was found in right 4D length between women with and without ASDs (*p* > 0.10, *d* = 0.41; Fig. 1a). By contrast, the other digits revealed a main effect of sex (*F*(1,146) = 62.15, *p* < 0.001 for right 2D; *F*(1,146) = 78.59, *p* < 0.001 for left 2D; *F*(1,146) = 80.73, *p* < 0.001 for left 4D), but no significant interactions of group and sex and main effects of group were identified (*p* > 0.05 for each). The effect sizes (Cohen’s *d*) of group differences between men with and without ASDs were 0.10 for right 2D, 0.18 for left 2D, and 0.24 for left 4D, and the corresponding differences between women with and without ASDs were 0.13, 0.16, and 0.34 [46]. Collectively, an interaction of group and sex was only found for the right 4D, while only sex effects were found for right 2D, left 2D, and left 4D.

**Table 2:** Digit length and 2D:4D ratios in each group

	Men				Women			
	ASDs	TD	ASDs vs TD		ASDs	TD	ASDs vs TD	
	Mean ± SD	Mean ± SD	*p*	*d*	Mean ± SD	Mean ± SD	*p*	*d*
Digit length								
Right 2D	7.21 ± 0.36	7.17 ± 0.43		0.10	6.61 ± 0.42	6.66 ± 0.39		0.13
Right 4D	7.68 ± 0.33	7.53 ± 0.45	†	0.37	6.82 ± 0.42	7.00 ± 0.44		0.41
Left 2D	7.24 ± 0.35	7.17 ± 0.41		0.18	6.56 ± 0.43	6.62 ± 0.37		0.16
Left 4D	7.67 ± 0.36	7.55 ± 0.57		0.24	6.84 ± 0.44	6.99 ± 0.44		0.34
2D:4D ratio								
Right	0.939 ± 0.029	0.952 ± 0.026	*	0.48	0.970 ± 0.035	0.953 ± 0.032	*	0.52
Left	0.944 ± 0.029	0.950 ± 0.029		0.21	0.963 ± 0.047	0.948 ± 0.033		0.41
Right minus left	−0.005 ± 0.021	0.003 ± 0.027		0.32	0.008 ± 0.035	0.005 ± 0.029		0.10

Digit length and 2D:4D ratios for each group are shown as mean ± SD. Digit lengths are shown in cm. *p* values of post hoc pair-wise comparisons and Cohen’s *d* values of comparison between ASDs and TD for each sex are presented

†*p* < 0.10; *p < 0.05

Regarding the 2D:4D ratios, two-way ANOVAs examining the right 2D:4D ratios according to group and sex revealed a significant interaction between sex and group (*F*(1,146) = 9.11, *p* = 0.003) and a main effect of sex (*F*(1,146) = 9.71, *p* = 0.002), but there was no significant main effect of group (*F*(1,146) = 0.16, *p* = 0.686). Post hoc pair-wise comparisons with the Bonferroni correction revealed that men with ASDs showed significantly lower right 2D:4D ratios versus those of TD men (*p* < 0.05, *d* = 0.48), and women with ASDs showed significantly higher 2D:4D ratios versus those of TD women (*p* < 0.05, *d* = 0.52; Fig. 1b). In contrast to the right 2D:4D ratio results, the two-way ANOVA results for the left 2D:4D and right–left 2D:4D ratios revealed no significant interactions between sex and group (*F*(1,146) = 3.25, *p* = 0.073 for left 2D:4D ratios; *F*(1,146) = 1.24, *p* = 0.267 for right–left 2D:4D ratios), main effect of group (*F*(1,146) = 0.79, *p* = 0.374 for left 2D:4D ratios; *F*(1,146) = 0.37, *p* = 0.546 for right–left 2D:4D ratios), or main effect of sex (*F*(1,146) = 2.15, *p* = 0.144 for left 2D:4D ratios; *F*(1,146) = 2.51, *p* = 0.115 for right–left 2D:4D ratios). Effect sizes (Cohen’s *d*) of group differences between men with and without ASDs were 0.21 for left 2D:4D and 0.32 for right–left 2D:4D, and those between women with and without ASDs were 0.41 for left 2D:4D and 0.10 for right–left 2D:4D. Collectively, women with ASDs exhibited higher right 2D:4D ratios relative to TD women, whereas men displayed lower right 2D:4D ratios relative to TD men. However, none of the group effects was found for either the left or right–left 2D:4D ratios.

### Relationship between 2D:4D ratios and total AQ scores

We then conducted correlation analyses between total AQ scores and digit length and total AQ scores and 2D:4D ratios separately for each group. Table 3 summarizes the results. We did not find a significant correlation between AQ total scores and digit length or 2D:4D ratios in any group.

**Table 3:** Correlation between AQ and digit length or 2D:4D ratios in each group

	Men				Women			
	ASDs		TD		ASDs		TD	
	*r*	*p*	*r*	*p*	*r*	*p*	*r*	*p*
Digit length								
Right 2D	−0.28	0.104	0.02	0.868	0.25	0.338	−0.03	0.842
Right 4D	−0.15	0.384	0.01	0.942	0.17	0.503	−0.01	0.951
Left 2D	−0.24	0.168	−0.02	0.898	0.21	0.430	0.06	0.684
Left 4D	−0.16	0.366	0.04	0.762	−0.05	0.861	0.01	0.936
2D:4D ratio								
Right	−0.21	0.215	0.03	0.800	0.12	0.657	−0.03	0.817
Left	−0.13	0.474	−0.12	0.369	0.27	0.296	0.06	0.643
Right minus left	−0.13	0.470	0.15	0.244	−0.24	0.349	−0.10	0.442

*r* and *p* values for the correlation analysis are shown for each group. Note that there were no significant correlations for any of the groups

### Do age and FSIQ explain the sex-dependent difference between the right 2D:4D ratio and autistic traits/ASDs?

Previous studies have suggested that 2D:4D ratios positively correlate with age [19, 29], and individuals with ASDs and severe mental retardation tend to exhibit lower 2D:4D ratios [27]. Therefore, we sought to determine whether the sex-dependent associations between the right 2D:4D ratios are related to age or FSIQ. The correlation analysis between right 2D:4D ratios and age revealed no significant correlations in men or women (*r*(92) = −0.10, *p* = 0.346 for men; *r*(72) = 0.04, *p* = 0.728 for women). Furthermore, when we excluded the effect of age, an analysis of covariance (ANCOVA) revealed a significant interaction between group and sex (*F*(1,163) = 9.01, *p* = 0.003). A post hoc pair-wise comparison with the Bonferroni correction showed significant (*p* < 0.05) group differences in both men and women. Therefore, sex-dependent group differences in the right 2D:4D ratios cannot be explained by age. Correlation analysis between the right 2D:4D ratio and FSIQ in the ASD group revealed a significant correlation in men but not in women (*r*(33) = 0.34, *p* = 0.046 for men with ASDs; *r*(15) = 0.36, *p* = 0.163 for women with ASDs). However, the correlation coefficients were similar between men and women, and no difference was found between the two slopes (*t*(48) = 0.65, *p* = 0.516). Therefore, it is unlikely that the sex-dependent association between the right 2D:4D ratio and ASDs or autistic traits can be explained by FSIQ.

## Discussion

### Sex-dependent association between the right 2D:4D ratio and ASDs

We confirmed that men with ASDs exhibited lower right 2D:4D ratios compared to TD men. These results suggest that high prenatal testosterone (relative to estrogen) is one of the risk factors for ASDs in Japanese men; thus, they support the EMB theory. Unexpectedly, our study also showed that women with ASDs displayed higher right 2D:4D ratios compared to TD women. This indicates that prenatal sex hormone activity can be a risk factor for Japanese women with ASDs. In contrast, we found no significant correlation between 2D:4D ratios and AQ total score. These results are consistent with previous meta-analyses [16, 17] and suggest that prenatal sex hormone activity affects the occurrence of ASDs, irrespective of individual autistic traits.

Because the 2D:4D ratio is an indirect measure of prenatal sex hormone activity, other confounding factors should be considered. Recent studies have found a correlation between 2D:4D ratios and chronological age in children both with and without ASDs [19, 29], and a longitudinal study showed that 2D:4D ratios increase throughout development in TD children [47]. Thus, postnatal factors may also alter the 2D:4D ratio. With respect to postnatal factors, Bloom et al. proposed that the lower 2D:4D ratios in subjects with ASDs reported in previous studies result from delayed skeletal maturation relative to TD subjects [19]. They measured the length of the phalanges and metacarpal bones of the left hands of children aged between 4 and 8 years with ASDs and demonstrated that the left 2D:4D ratios were positively correlated with skeletal age measured using Tanner–Whitehouse 3 (TW3) ratings [19]. However, all participants in the present study were older than 15, so the 2D:4D ratios in the present study would be less affected by differences in skeletal developmental stages relative to studies involving young children. Furthermore, if the sex-dependent associations between the 2D:4D ratio and the occurrence of ASDs result from differences in skeletal development, similar patterns should be found in the left 2D:4D ratios. However, we only observed sex-dependent group differences for the right 2D:4D ratios, which are predominantly affected by prenatal sex hormone activity [12, 45]. Therefore, it is unlikely that lower 2D:4D ratios in men with ASDs and higher 2D:4D ratios in women with ASDs reflect differences in skeletal maturation between groups.

Postnatal testosterone exposure has also been proposed to affect 2D:4D ratios [29, 48]. Previous studies reported elevated rates of testosterone-related disorders in women with autism [49] and elevated postnatal blood testosterone levels in women with ASDs [28, 50, 51]. If the effect of postnatal testosterone exposure is similar to that of prenatal testosterone, excessive exposure should result in lower 2D:4D ratios in women with ASDs. In contrast, we found higher 2D:4D ratios in women with ASDs, making it unlikely that those higher 2D:4D ratios were caused by postnatal testosterone exposure.

Another possible confounding factor is 2D:4D ratio fluctuation over the menstrual cycle [52]. Mayhew et al. reported that the right 2D:4D ratio of TD women who took oral contraceptives fluctuated over their menstrual cycle within 0.01, but no fluctuation was found for TD women who did not take oral contraceptives [52]. However, none of women with ASDs and just 1 of the 57 TD women used oral contraceptives in the present study. Furthermore, we found that the difference between the right 2D:4D ratio of ASD and TD women was 0.017 (greater than 0.01). Therefore, it is unlikely that the difference in the right 2D:4D ratio between ASD and TD women is associated with their menstrual cycles.

We can rule out the possibility that higher 2D:4D ratios in women with ASDs are caused by differences in skeletal developmental stages, postnatal testosterone exposure, or menstrual cycle. Therefore, it is reasonable to conclude that the higher right 2D:4D ratios reflect prenatal sex hormone activity differences in Japanese women with ASDs.

### Possible mechanisms underlying the sex-dependent difference in the right 2D:4D ratio as a risk factor for ASDs

Why do women with ASDs have higher right 2D:4D ratios than TD women, unlike men with ASDs? One possibility is higher prenatal testosterone activity. Indeed, several studies have reported an association between prenatal testosterone levels in mothers and ASDs or higher autistic traits in daughters [49, 53, 54]. For instance, mothers of women with ASDs exhibited an increased rate of testosterone-related medical conditions [49]. Furthermore, the daughters of hyperandrogenic mothers with polycystic ovarian syndrome (PCOS) tend to have higher AQ scores and systemizing quotient scores as well as lower empathy quotient scores. Interestingly, the same study also showed elevated amniotic fluid testosterone levels and higher right 2D:4D ratios in daughters of mothers with PCOS compared to those without [53]. These findings imply that high testosterone levels in mothers can lead to both ASDs and higher right 2D:4D ratios in daughters.

How can high testosterone in mothers cause higher right 2D:4D ratios in their daughters? One possibility is altered growth of soft tissue (e.g., fingertip fat) induced by excessive prenatal testosterone exposure. Previous animal studies demonstrated that high testosterone activity affects the growth of skeletal and soft tissue in index and annular fingers [15, 55]. In a rat study, Zheng et al. showed that higher activity of androgen relative to the estrogen receptor led to abnormal phalange growth. Injecting the dam with dihydrotestosterone induced annular finger elongation and a lower 2D:4D ratio for female offspring [15]. In contrast, Abbott et al. examined the effect of injecting testosterone into the dam during gestation on the 2D:4D ratio in rhesus monkeys. They measured phalange length or joint spaces and length from the basal crease to the digit tip, which accounted for both skeletal and soft tissue. They showed that female offspring of dams injected with testosterone had elongated index fingers and an increased 2D:4D right ratio when they measured digit length from the skin, while no abnormality was observed in skeletal tissue [55]. Their results suggest that female fetuses exposed to high prenatal testosterone have abnormal soft tissue growth (i.e., fat accumulation in the right index fingertip) and an increased right 2D:4D ratio for rhesus monkeys. These findings are notable since they are in non-human primates.

In human studies of 2D:4D ratio, the index and annular finger lengths can be measured in several ways, such as the length from the basal crease to fingertips from photocopies or direct measurement, which includes both soft and skeletal tissues. Further, 2D:4D ratio are also measured by radiograph which can evaluate phalange length. Several radiograph studies have shown a sex difference in the 2D:4D ratio for TD individuals [56–58], indicating that skeletal tissue contributes to this ratio. In addition, several studies have suggested that fingertip fat also contributes to the sex difference in 2D:4D ratios for TD individuals [59–62]. For instance, some studies showed larger 2D:4D ratios for indirect measurement (i.e., photocopies) compared to direct measurement (i.e., direct measurement of participants hands) [59, 60]. Manning et al. proposed that sex differences in fingertip fat shape might explain differences between these two measurements. Manning et al. previously showed that the 2D:4D ratio measured on photocopies showed a larger sex difference than phalanges assessed with radiographs [61]. Furthermore, a recent study demonstrated that the 2D:4D ratio of phalanges in women is not related to various indices including anthropometric, behavioral, and nutritional variables. Vehmas proposed the possibility that these features might be associated with soft tissue rather than bone length [62]. Based on these findings, we speculate that the higher 2D:4D ratio for women with ASDs might be due to alterations in fat tissue.

Collectively, it is possible that a higher 2D:4D ratio for women with ASDs might reflect altered fingertip fat caused by higher prenatal testosterone exposure of maternal origin. Therefore, we speculate that high prenatal testosterone exposure affects different tissues in each sex, such as soft tissue for women and skeletal tissue for men. Alternatively, high prenatal testosterone exposure could affect the same tissues differently in men and women. An examination of skeletal and soft tissue would be necessary to elucidate sex-difference abnormalities in right 2D:4D ratios in subjects with ASDs.

### Factors that explain compatibility between previous findings and the present study

Although we found higher 2D:4D ratios in women with ASDs versus TD women, previous studies contradict this result [28, 29, 63]. Similar to studies by Hauth et al. and Bejerot et al., Lai et al. reported no significant difference in the right 2D:4D ratio between women with and without ASDs in magnetic resonance imaging studies [63]. These contradictory results can be explained by race differences or by confounding factors such as skeletal maturation or FSIQ. For instance, Hauth et al. reported no significant difference in the mean 2D:4D ratios between 38 girls with ASDs and 95 TD girls. However, 2D:4D ratios were correlated with skeletal age in children with ASDs [29], so the failure to find group differences in 2D:4D ratios in the Hauth et al. study may have been caused by individual variability in the skeletal developmental stages. Bejerot et al. also reported no difference in 2D:4D ratios between 24 women with ASDs and 25 TD women. However, the mean right 2D:4D ratios were slightly higher in women with ASDs (0.98) relative to TD women (0.97). In addition, they did not examine the IQs of ASD participants, and the ASD group included a large number of poorly educated women (<9 or 12 years of schooling) compared to the TD group [29]. Previous studies have suggested that individuals with ASDs and severe mental retardation tend to display lower 2D:4D ratios [27], and we found a marginally significant correlation between FSIQ and right 2D:4D ratios in individuals with ASDs. One possible explanation for the failure of Bejerot et al. to find a significant difference is that the 2D:4D ratios in the ASD group were reduced by low IQ rather than ASD in women with ASDs.

In addition to the confounding factors described above, race differences might be a cause of contradictory results among reports. One possibility is that vulnerability to sex hormone exposure differs between races. Previous studies have shown an association between ASDs (diagnosis or autistic features in typical development) and various genetic predispositions related to sex hormone synthesis, metabolism, or receptors [64–67]. For example, the number of CAG repeats in androgen receptor genes [65] is believed to be related to the 2D:4D ratio [14]. Such genetic predispositions frequently differ among ethnic groups [68, 69]. For instance, Japanese TD individuals tend to have a shorter CGC repeat in the androgen receptor gene and thus higher androgen receptor activity compared to German (Caucasian) individuals [69]. In addition, a previous study suggested that testosterone levels in umbilical cord blood were higher for Asian babies in China than Caucasian babies in the USA [33]. Based on these studies, we can speculate that Japanese individuals tend to have a higher risk of maternal testosterone exposure than Caucasian individuals. This could contribute to the discrepant results of previous and present studies.

### Limitations and further study

Our results should be considered in the context of five limitations. First, we found no significant sex difference in the right 2D:4D ratio for the TD group. One possible reason is that our inclusion criterion for the TD group (AQ < 20) affected the sex difference in the TD group. To address the issue, our findings should be replicated with large samples that include TD subjects with higher autistic traits. Second, the 2D:4D ratio is an indirect measure of prenatal sex hormone activity, so we could not identify the root cause(s) of the different 2D:4D ratios between women with and without ASDs. Direct measures such as sex hormone levels in amniotic fluid or maternal blood or the study of the genetic predispositions would provide more direct and robust evidence. In addition, radiographic examination of phalange length should allow us to determine whether the increased right 2D:4D ratio for Japanese women with ASDs was due to soft or skeletal tissue alternations. Furthermore, it is unknown which mechanism affects abnormal fat growth in the fingertips of female offspring exposed to higher testosterone in utero (e.g., gene expression or activation of androgen receptors). Genetic or molecular studies of non-human primates are necessary to elucidate which mechanisms influence 2D:4D ratios in subjects with ASDs. Third, we only examined the 2D:4D ratios in Japanese individuals; thus, the examination of both prenatal sex hormone levels and sex hormone-related genetic predispositions across multiple races would be necessary to explain race-based differences. Fourth, it has been suggested that postnatal sex hormones also play an important role in modulating social behaviors [10], and responses to testosterone administration, such as empathy or cooperation, differ according to the responder’s 2D:4D ratio [70–72]. Thus, prenatal sex hormone activity can be considered to characterize the sensitivity to postnatal testosterone in terms of social cognition. Further study examining both prenatal and postnatal testosterone is necessary to understand the etiology and pathophysiology of women with ASDs. Fifth, we recruited ASD participants through the Department of Neuropsychiatry at the University of Fukui Hospital, which might have introduced a sampling bias. Our findings should be confirmed in a multicenter study.

## Conclusions

In the present study, we found that right 2D:4D ratios were lower in Japanese men with ASDs compared to TD Japanese men. Conversely, Japanese women with ASDs exhibited higher right 2D:4D ratios than TD Japanese women. It has been proposed that high prenatal testosterone leads to lower 2D:4D ratio; however, a recent animal study showed that testosterone injections given to dams induced higher right 2D:4D ratios, especially for female offspring. Therefore, the results of the present study suggest that high prenatal testosterone could be a risk factor both for Japanese men and women with ASDs, elucidating one potential etiology of ASDs in women.

## Abbreviations

ASDs: Autism spectrum disorders
TD: Typically developed
AQ: Autism-spectrum quotient
EMB theory: Extreme male brain theory
FSIQ: Full-scale IQ
SNP: Single nucleotide polymorphisms
